# A Small-Scale shRNA Screen in Primary Mouse Macrophages Identifies a Role for the Rab GTPase Rab1b in Controlling *Salmonella* Typhi Growth

**DOI:** 10.3389/fcimb.2021.660689

**Published:** 2021-04-07

**Authors:** Virtu Solano-Collado, Rosa Angela Colamarino, David A. Calderwood, Massimiliano Baldassarre, Stefania Spanò

**Affiliations:** ^1^Institute of Medical Sciences, University of Aberdeen, Aberdeen, United Kingdom; ^2^Department of Pharmacology, Yale University School of Medicine, New Haven, CT, United States; ^3^Department of Cell Biology, Yale University School of Medicine, New Haven, CT, United States

**Keywords:** host-defense, *Salmonella* Typhi, Rab GTPases, Rab1b, shRNA screen, macrophages

## Abstract

*Salmonella* Typhi is a human-restricted bacterial pathogen that causes typhoid fever, a life-threatening systemic infection. A fundamental aspect of *S*. Typhi pathogenesis is its ability to survive in human macrophages but not in macrophages from other animals (i.e. mice). Despite the importance of macrophages in establishing systemic *S*. Typhi infection, the mechanisms that macrophages use to control the growth of *S*. Typhi and the role of these mechanisms in the bacterium’s adaptation to the human host are mostly unknown. To facilitate unbiased identification of genes involved in controlling the growth of *S*. Typhi in macrophages, we report optimized experimental conditions required to perform loss-of function pooled shRNA screens in primary mouse bone-marrow derived macrophages. Following infection with a fluorescent-labeled *S*. Typhi, infected cells are sorted based on the intensity of fluorescence (i.e. number of intracellular fluorescent bacteria). shRNAs enriched in the fluorescent population are identified by next-generation sequencing. A proof-of-concept screen targeting the mouse Rab GTPases confirmed Rab32 as important to restrict *S*. Typhi in mouse macrophages. Interestingly and rather unexpectedly, this screen also revealed that Rab1b controls *S*. Typhi growth in mouse macrophages. This constitutes the first report of a Rab GTPase other than Rab32 involved in *S*. Typhi host-restriction. The methodology described here should allow genome-wide screening to identify mechanisms controlling the growth of *S*. Typhi and other intracellular pathogens in primary immune cells.

## Introduction

Upon bacterial invasion, cells of the innate immune system such as macrophages act as the first line of defense to control the infection. Macrophages are equipped with an array of antimicrobial factors, allowing them to efficiently eliminate invaders (Weiss and Schaible, 2015). However, some bacterial pathogens have evolved mechanisms to resist the attack by macrophages and adopt an intracellular lifestyle, persisting inside these cells. The Gram-negative pathogen *Salmonella enterica*, upon internalization by a host cell, establishes an intracellular niche by creating a specialized compartment known as *Salmonella*-containing vacuole where the bacterium survives and replicates. The capability of *Salmonella* to exploit and survive within macrophages is essential for its pathogenesis and for the establishment of systemic infection (Fields et al., 1986). This intracellular survival is made possible by the action of type-III secretion systems (T3SS) that inject effector proteins into target cells to manipulate host pathways. *Salmonella* encodes two T3SS within the pathogenicity islands 1 (SPI-1) and 2 (SPI-2). Some of these effectors block specific macrophage killing mechanisms while others facilitate bacterial invasion and create a habitable intracellular niche (Galán et al., 2014; Spanò et al., 2016; Jennings et al., 2017).

The species *Salmonella enterica* comprises several serovars and while most can infect a broad range of mammalian species, others are host specific or host-restricted. *Salmonella enterica* serovar Typhi (*S*. Typhi) is a host-restricted serovar that only infects humans (Dougan and Baker, 2014) and causes the life-threatening condition typhoid fever, resulting in between 11.9 and 26.9 million annual cases around the globe (Gibani et al., 2018). The failure of this pathogen to infect other species is partially due to its inability to overcome a host defense mechanism, which depends on the Rab GTPase Rab32 and its nucleotide exchange factor Biogenesis of Lysosome-related Organelles Complex-3 (BLOC-3) (Spanò and Galán, 2012; Spanò et al., 2016). This pathway is efficiently disarmed by the broad-host serovar *S*. Typhimurium through the delivery of two T3SS effectors: GtgE and SopD2. Both act directly on Rab32, GtgE is a protease and SopD2 is a GTPase activating protein (Spanò et al., 2016). In *S*. Typhi GtgE is absent while SopD2 is a pseudogene.

Rab GTPases are key regulators of vesicle trafficking between organelles and an interface between the host cell and the internalized pathogen. Thus, it is not surprising that successful intracellular pathogens use the manipulation of these proteins as a mechanism to survive within their eukaryotic hosts (Bakowski et al., 2010; Seto et al., 2011; Hardiman et al., 2012; McGourty et al., 2012; Stein et al., 2012; Spanò and Galán, 2018). Despite efforts to understand the nature of *S*. Typhi host-restriction, most of our knowledge of *Salmonella* interactions with Rab GTPases is extrapolated from *S*. Typhimurium studies, and many questions as to how macrophages deal with *S*. Typhi remain open. In this study, using a loss-of-function shRNA-based approach, we have performed a screen in mouse bone-marrow derived macrophages and identified Rab1b as a factor controlling *S*. Typhi growth in mouse macrophages.

## Materials and Methods

### Bacterial Strains and Mammalian Cell Culture

The *S*. Typhi wild-type ISP2825 (Galán and Curtiss, 1991) and the *S*. Typhi *glmS::Cm::mCherry* (*S*. Typhi::*mCherry*) (Baldassarre et al., 2021) strains have been described previously.

*Salmonella* can induce caspase 1-dependent macrophage death (Brennan and Cookson, 2000). To avoid this, bone-marrow-derived macrophages (BMDMs) were isolated from caspase 1-deficient mice (casp1-/-) and differentiated as described before (Weischenfeldt and Porse, 2008).

Immortalized bone-marrow-derived macrophages (iBMDMs), RAW264.7 and HEK293T cell lines were routinely maintained in DMEM high glucose 2 mM glutamax (Thermo Fisher) and 10% FBS (Gibco).

### Infection of Macrophages With Salmonella Typhi for Intracellular Survival Assays (CFU Assay)

Overnight cultures of *Salmonella* were diluted 1/20 in LB broth containing 0.3 M NaCl and grown at 37°C to an OD600 of 0.9. Cells were washed twice with HBSS (Thermo Fisher) and infected with *S*. Typhi at the indicated multiplicity of infection. One hour post-infection, cells were washed twice with HBSS and incubated in growth medium supplemented with 100 µg/ml gentamicin for 30 min to kill extracellular bacteria. Then, medium was replaced with growth medium containing 5 µg/ml of gentamicin to avoid cycles of reinfection. At the indicated times-post infection, cells were washed twice with PBS, lysed in 1 ml 0.1% sodium deoxycholate (Sigma) in PBS and intracellular bacteria counted by plating serial dilutions on LB-agar plates.

### *Salmonella* Typhi Infection of Macrophages for Flow-Cytometry and Fluorescence Microscopy

To determine the optimal multiplicity of infection (MOI), 6x10^5^ BMDMs were plated on 6-well plates and infected with different amount of fluorescent bacteria (from MOI 5 to 30). One hour-post infection, medium was replaced with medium containing 100 µg/ml gentamicin for 30 minutes and cells were collected by incubating with cold Versene (Gibco) for 5 minutes, fixed with 4% paraformaldehyde (PFA) for 10 minutes at room temperature and analyzed using the Fortessa flow cytometer. In parallel, 1x10^5^ BMDMs were plated on 24-well plates and infected as before. Cells were then fixed with 4% PFA and visualized with a Zeiss Imager M1 fluorescence microscope. For quantification of the number of bacteria per cell, a minimum of 72 infected cells were analyzed per condition and the results plotted with Prism 8.

To confirm that shRNA-mediated gene knockdown resulted in a measurable phenotype, 4x10^6^ BMDMs were transduced with shRNAs targeting HPS-1 or scrambled sequence and plated on 6-well plates (6x10^5^ cells/well; flow cytometry) or 24-well plates (1x10^5^ cells/well; fluorescence microscopy and CFU assays). After selection with puromycin, transduced cells were infected with *S*. Typhi::*mCherry* and samples at 1.5, 5 and 24 hours post-infection were analyzed by flow cytometry and fluorescence microscopy as described above. For quantification of the number of bacteria per cell, a minimum of 100 cells were used per condition and the results plotted with Prism 8.

### RNA Isolation and RT-qPCR

Total RNA from eukaryotic cells was isolated using the RNeasy mini kit (QIAGEN). RNA was transcribed using the iScript reverse transcriptase (BioRad) and transcript levels were determined using the Takyon SYBR MasterMix (Eurogentec) and the StepOnePlus real-time PCR system (Applied BioSciences). Primers are listed in **Table S5**.

### Generation of Rab GTPases shRNA Library and Screening

The mouse Rab GTPases shRNA library (317 shRNAs) was extracted from The Mission mouse shRNA library generated by The RNAi consortium (Sigma Aldrich) and prepared as described before (Shi et al., 2016). The shRNA targeting the HPS-1 gene as well as the packaging plasmids pCMV-VSV-G and pCMV-dR8.91 were obtained from Sigma Aldrich. The plasmids encoding the shRNA sequences were pooled in equal concentrations and mixed with pCMV-VSV-G (1:0.1) and pCMV-dR8.91 (1:1) to generate lentiviral particles. HEK293T cells were transfected with the plasmid mixture and polyethyleneimine as transfecting reagent. Fifty-two hours post-transfection, the lentivirus particles were collected from cell supernatants.

Three independent experiments were performed and sorted independently. For each, a total of 1x10^7^ BMDMs were transduced at day 4 after isolation for 24 hours and selected in puromycin (5 µg/ml) for 48 hours. Cells were then infected with *S*. Typhi::*mCherry* at an MOI of 10. At 1.5 and 24 hours-post infection, cells were processed for flow cytometry as described above. A total of 50,000 cells (N and M populations) or 17,000 to 20,000 cells (H population) were sorted in a BD Influx BSLII Sorter (Iain Fraser Cytometry Centre, University of Aberdeen) (**Figure S3**). Genomic DNA (gDNA) was isolated using the DNeasy Blood & Tissue kit (QIAGEN) following the instructions of the manufacturer with the following modifications: after cell lysis, samples were incubated at 90°C for 1 hour to reverse PFA modification of DNA. The resulting gDNA was used as template for PCR amplification of the shRNA-encoding regions using the Fw-NGS and the Rev-NGS primers (IDT Technologies) and the Phusion High Fidelity DNA polymerase (NEB). Three PCRs per condition were performed and the amplicons combined. Primer sequences are listed in **Table S5**. A second PCR was performed to attach indexes and Illumina adaptors and the PCR amplicon was sequenced on the NextSeq500 v2 instrument (Centre for Genome-Enabled Biology and Medicine (CGEBM), University of Aberdeen).

### Data Analysis

The analysis of the data involved background correction and scoring. Firstly, the proportion of reads relative to total reads of each shRNA was obtained using the BBDUK software (CGEBM, University of Aberdeen). shRNAs with value “0” in 2 out of the 3 experiments in the plasmid library or controls (non-sorted cells) at either 1.5 and 24 hours-post infection were excluded from the analysis (**Table S2**). Then, the proportion of reads of each shRNA within the high fluorescence population (H) relative to the total proportion of reads (H, M and N populations) was calculated as well as the average and standard deviation of the population. Finally, the z-score for each shRNA was calculated as z = (x-μ)/σ, where x is the value obtained of each shRNA (proportion of reads of each shRNA within the high fluorescence population (H) relative to the total reads (N+M+H), μ is the population mean and σ is the standard deviation of the population (**Table S3**). True hits were defined as those for which at least 2 shRNAs had a z-score ≥1.18 based on the scores obtained for internal positive controls (**Table S4**).

### Validation of Candidate Genes by Individual Gene Knockdown and Intracellular Survival Assay

iBMDMs were transduced with lentivirus encoding shRNA to knockdown individually candidate genes 6 days before infection. Twenty-four hours after transduction, cells were incubated with 5 µg/ml puromycin for 2-4 days. *S*. Typhi infection assays were carried out as described before.

## Results

### shRNA-Mediated Knockdown in Bone-Marrow Derived Macrophages

**Figure 1:**
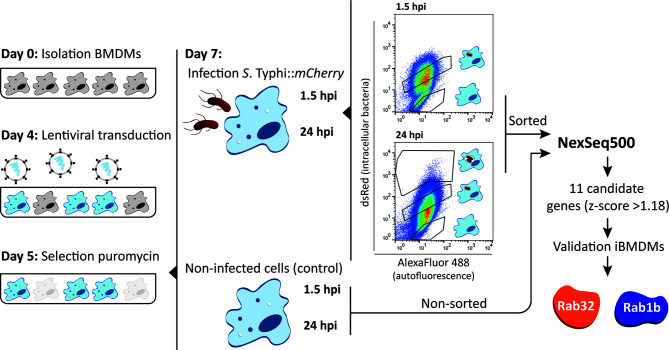
shRNA screening in BMDMs. Schematic representation of the experimental approach of the shRNA screening in BMDMs. BMDMs were isolated from casp1-/- mice and transduced at day 4 after isolation. After selection with puromycin for 48 hours, cells were infected with *S*. Typhi::*mCherry*. At 1.5 and 24 hours post-infection cells were fixed with 4% PFA and the different sub-populations sorted based on dsRed fluorescence (gates for the different sub-populations indicated). Genomic DNA from sorted and non-sorted cells (control) was isolated, the region encoding the shRNA amplified by PCR and the amplicons sequenced by next-generation sequencing (NextSeq500). As a result of the screen, 11 candidate genes were identified based on a z-score >1.18 and hits validated using shRNAs to knockdown the individual genes.

Pooled shRNA screens offer unbiased approaches to identify genes important for specific biological processes (Silva et al., 2008; Carette et al., 2011). As illustrated in **Figure 1**, we designed an shRNA screening strategy to identify macrophage proteins whose knockdown facilitated *S*. Typhi growth. The approach requires efficient delivery of the shRNA library into primary macrophages to generate a pool of knockdown cells coupled with an effective sorting strategy to collect cells that failed to clear the invading bacteria. As described below we optimized each of these steps.

**Figure 2:**
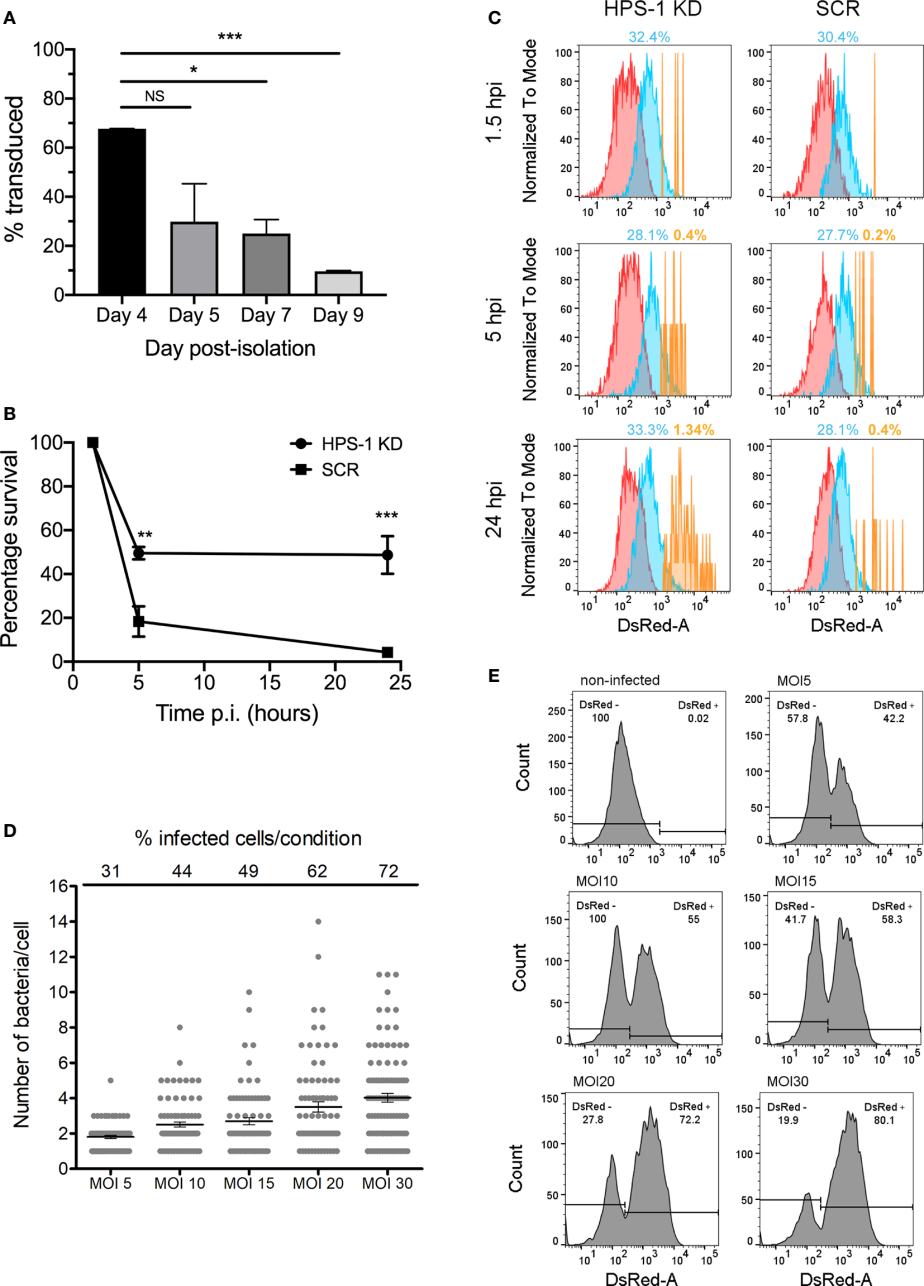
shRNA-mediated knockdown in BMDMs. **(A)** BMDMs were transduced at different days post-isolation. After incubation with 5 μg/ml puromycin for 48 hours, cell viability was evaluated with alamar blue assay. Percentage of survival are: Day 4 67.72%, Day5 26.84%, Day 7 25.02%, Day 9 9.63%. Values are mean ± SEM of two independent measurements. NS, non-significant; *P < 0.05; **P < 0.01; ***P < 0.005. **(B)** BMDM cells were transduced with lentiviral particles encoding a non-target scrambled sequence (SCR) or encoding a sequence targeting HPS-1 (HPS-1 KD). After selection with 5 µg/ml puromycin, cells were infected with *S*. Typhi wild-type, lysed at three times post-infection (1.5, 5 or 24 h) and CFUs were enumerated. Values are means ± SEM of two independent experiments performed in triplicate. *P* values were determined by the Student´s *t* test and are indicated when *P* < 0.05. **(C)** BMDMs HPS-1 Knockdown (HPS1 KD) or control (Scrambled shRNA; SCR) were infected with *S*. Typhi::*mCherry* (MOI 10). At different times post-infection (1.5, 5 and 24 hours post-infection), cells were fixed with 4% PFA and analyzed by flow cytometry. The three subpopulations non-infected (N) (red), 1-3 bacteria (M) (blue) and >3 bacteria (H) (yellow) are shown and the percentage of the M and H populations are indicated. **(D, E)** BMDMs were infected with *S*. Typhi::*mCherry* at different multiplicity of infection (MOI). After 1.5 hours-post infection, cells were fixed with 4% PFA and analyzed by fluorescence microscopy **(D)** or flow cytometry **(E)** to determine the percentage of infected cells and the average number of bacteria per cell. For quantification of the number of bacteria per cell for fluorescence microscopy, 85 (out of 272 total; MOI 5), 83 (out of 190 total; MOI 10), 72 (out of 147 total; MOI 15), 80 (out of 128 total; MOI 20) and 88 (out of 122 total; MOI 30) infected cells were analyzed.

Loss of function screenings in primary macrophages require optimization of conditions to achieve efficient and reproducible gene knockdown within the macrophage lifespan and without affecting their final differentiation. Although several methods to introduce exogenous DNA in primary murine macrophages have been reported (Thompson et al., 1999; Guiet et al., 2012), lentiviral systems have been shown to be the most efficient method (Naldini et al., 1996; Schroers et al., 2000). While Zeng et al., have shown that culturing macrophages for 8 days *in vitro* increased the transduction efficiency using HIV-based vectors (Zeng et al., 2006) others recommended earlier stages of differentiation for efficient transduction (Miller and Blystone, 2015). In order to determine, under our experimental conditions, the optimal post-isolation day to transduce BMDMs, we used a lentivirus encoding a puromycin resistance marker and transduced BMDMs that were in culture from day 4 to day 9 of differentiation. Transduced cells were selected with 5 µg/ml puromycin for 48 hours and cell survival determined by alamar blue assay. The results obtained showed that at the early stages of the differentiation into macrophages (day 4), cells are transduced more efficiently than at later stages (days 7 to 9) (**Figure 2A**). Importantly, the level of transduction achieved (≈60%) ensured that the probability of having a cell infected by more than one virus (i.e. more than one shRNA) is low.

We then confirmed that the viral transduction process did not affect the ability of mouse macrophages to control *S*. Typhi growth. Indeed, BMDMs transduced at day 4 with a virus encoding a non-targeting shRNA scrambled sequence (SCR) are still able to control *S*. Typhi growth (**Figure 2B**). Next, we knocked-down HPS-1 (≈95% knockdown; **Figure S1**) in BMDMs. HPS-1 is one of the two subunits of the BLOC-3 complex, which together with Rab32, controls *S*. Typhi growth in macrophages (Spanò and Galán, 2012; Baldassarre et al., 2021). We assessed *S*. Typhi intracellular survival in HPS-1-depleted macrophages using CFU assays (**Figure 2B**), flow cytometry (**Figure 2C**) and fluorescence microscopy (**Figure S2**). After infection with *S*. Typhi*::mCherry*, samples were analyzed at three different times post-infection: 1.5 (internalized bacteria), 5 and 24 hours. As shown in **Figure 2C**, at 24 hours post-infection, there is a population representing 1.34% of the HPS-1-depleted cells containing higher loads of intracellular bacteria, while this population only represents 0.4% of the control cells. Similarly, the analysis by fluorescence microscopy showed that the number of intracellular bacteria per cell significantly increased at late time points in HPS-1 knockdown cells but not in control cells (**Figure S2**).

Even though we cannot exclude subtle changes, these data confirmed that viral-delivered shRNAs per se do not affect the macrophages ability to restrict *S*. Typhi and that shRNA-dependent gene knockdown results in an assessable phenotype in BMDMs.

### Flow Cytometric Analysis of *S*. Typhi Infection in BMDMs

Our screening approach relies on an efficient flow cytometric method to detect and isolate cells that, upon gene silencing, are more permissive to *S*. Typhi growth. However, if a macrophage lacks a factor required to control the growth of *S*. Typhi, replication of the bacteria within the cell could ultimately lead to cell death and loss of a positive hit. To limit this, we have optimized the ratio of bacteria/cell during the infection (multiplicity of infection; MOI) in order to obtain a reasonable infection rate (50-60% infected cells) with low numbers of bacteria in each infected cell (2-3 bacteria per cell). For this, BMDMs were infected with different amounts of *S*. Typhi*::mCherry*, from MOI 5 to 30. One-hour post infection, cells were treated with gentamicin for 30 minutes to kill any extracellular bacteria, fixed and the percentage of infected cells, as well as the number of bacteria per cell, was determined by fluorescence microscopy (**Figure 2D**) and flow cytometry (**Figure 2E**). When cells were infected with an MOI of 5, the mean number of intracellular bacteria was 2 and the percentage of infected cells was around 30% (42.2% flow cytometry). In cells infected with an MOI of 10, the percentage of infected cells increased to 44% (55% flow cytometry) and only 9 out of 83 infected cells contained more than 4 intracellular bacteria. Higher MOI resulted in higher number of infected cells (from 42.2% to 80.1%) but the number of bacteria per cell increased notably as well, finding a considerable number of cells with more than 6 bacteria per cell with the highest MOI (**Figure 2D**). Therefore, all subsequent experiments were performed using an MOI of 10. In addition, these analyses allowed us to determine the sensitivity of the flow cytometer. Cells with as few as 1-2 fluorescent intracellular bacteria were detectable as an independent population easily distinguishable from non-infected cells (**Figure 2E**).

### shRNA Screen of Rab GTPases Involved in *S*. Typhi Growth Restriction in Mouse Macrophages

To validate our screening strategy and to identify novel Rab GTPases involved in restricting the growth of *S*. Typhi in macrophages, we performed a targeted loss-of-function shRNA screen in BMDMs using a custom-made pooled library (**Table S1**; summarized in **Figure 1**). We also included a validated shRNA targeting HPS-1 as an internal positive control.

Three independent screens were performed. For each one, 1x10^7^ BMDMs were transduced at 4 day after isolation with a pool of lentiviruses encoding the library and the positive control (see *Materials and Methods*). After 48 hours of selection with 5 µg/ml puromycin, transduced macrophages were infected with *S*. Typhi*::mCherry* at MOI 10. One hour after infection, the medium was replaced with fresh medium containing 100 µg/ml gentamicin for 30 min. At 1.5- and 24-hours post-infection, cells were lifted from the plates, fixed with 4% PFA and the different sub-populations of cells sorted based on red-fluorescence using an strategy that enabled enrichment of the very low abundant population containing more than 3 bacteria (an example is shown in **Figure S3**). Based on our fluorescence experiments, the sorted populations contains on average no bacteria (N), 1-3 bacteria (M) and >3 bacteria (H).

Once sorted, the genomic DNA from each cellular sub-population was isolated, the regions containing the shRNA sequences were PCR-amplified and the amplicons subjected to next-generation sequencing. Amplicons obtained from both the plasmid library and non-sorted cells were sequenced and used as controls to determine i) the abundance of each shRNA in the initial plasmid pool, ii) whether a particular hairpin present in the plasmid library was lost after transduction (absent from non-sorted 1.5 hours post-infection sample) and iii) whether a hairpin was lost over time independently of the infection (absent from non-sorted 24 hours post-infection sample) (**Figure 1** and **Table S2**).

Out of the 318 shRNAs that comprised the library, 7 shRNAs were not present or not detected by deep sequencing (0 reads in the plasmid library sample), 8 shRNAs were not present in non-sorted samples at either 1.5 or 24 hours and the reads for 2 shRNAs were “0” in all the sub-populations in at least one full experiment. All those shRNAs were not considered for further analyses (**Table S2**). Using these criteria none of the 56 genes included in the library were lost and at least 3 shRNAs per gene were included in the analysis. To obtain the list of candidate genes involved in *S*. Typhi growth-restriction and therefore those more abundant in the H population (**Figures 1** and **S3**), we first calculated for each shRNA the total proportion of reads (N, M and H) as well as the proportion of reads within the H sub-population. Then, we calculated the percentage of reads in H relative to total reads (N, M and H). Finally, we calculated the z-score for each individual shRNA (see *Materials and Methods*, **Figure S3B** and **Table S3**). By performing this analysis, the z-score values obtained for the positive controls were: HPS-1, z = 1.22 and Rab32 z = 1.18, z = 2.29, z = 0.63, z = -0.37 and z = -1.18). This variation is expected since not all shRNA sequences will result in knockdown. In view of the scores obtained for known positive hits, we opted for a less stringent strategy in order to minimize the chances of excluding positive candidates. Therefore, we selected 1.18 as threshold to call out hits and used the following criterion to generate a candidate gene list: 2 or more shRNAs with a z-score >1.18. Using this strategy we identified 11 candidate genes (≈20% of the total genes) that are listed in **Table S4**.

### Rab1b Is Important to Control *S*. Typhi Growth in Mouse Macrophages

**Figure 3:**
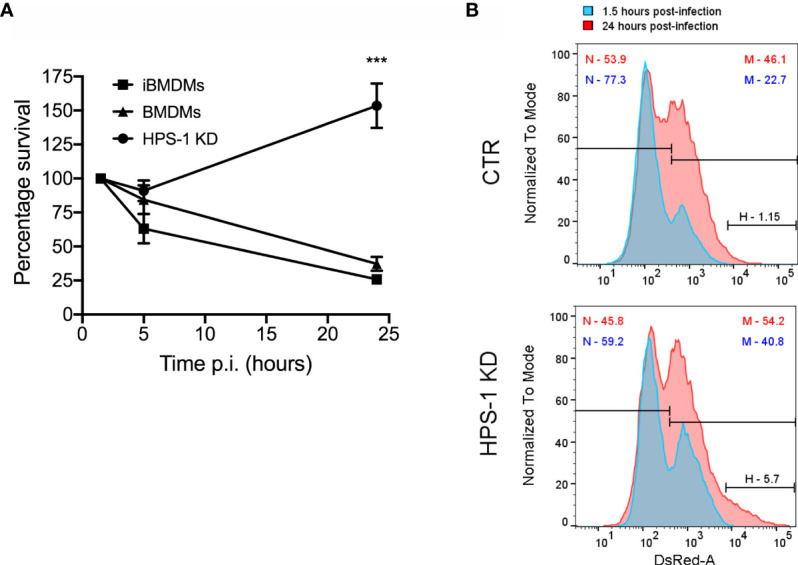
Depletion of HPS-1 allows *S*. Typhi to survive better in immortalized bone-marrow-derived macrophages (iBMDMs). **(A)** BMDMs, iBMDMs or iBMDMs HPS-1 knockdown (HPS-1 KD) were infected with *S*. Typhi wild-type (MOI 5). At the indicated times post-infection, cells were lysed and CFU enumerated. The CFUs obtained are normalized against the first time point 1.5 hpi, which is considered as 100% of uptake. Values are means ± SEM of two independent experiments performed in triplicates. *P* values were determined by the Student´s *t* test: ***, *P* < 0.005. **(B)** iBMDMs transduced with lentivirus encoding either non-targeting sequences (CTR) or an shRNA targeting HPS-1 (HPS-1 KD) were infected with *S*. Typhi::*mCherry*. At different times post-infection (1.5 or 24 hpi) cells were fixed and analyzed by flow cytometry. The abundance in percentage of the different subpopulations is indicated.

To prove the reproducibility of the data independently of the cell model, we used immortalized BMDMs (iBMDMs) to validate our hits. Additionally, this allowed us to reduce the use of animals in line with the principles of the 3Rs (Replace, Reduce, Refine). We first confirmed that iBMDMs behave similarly to BMDMs in their ability to kill *S*. Typhi (**Figure 3A**). We also confirmed that HPS-1 depleted iBMDMs are more permissive to *S*. Typhi growth by CFU assays and flow cytometry (**Figures 3A, B**). The results obtained showed that iBMDMs could be used as a suitable cellular model to validate the candidates from the shRNA screen.

**Figure 4:**
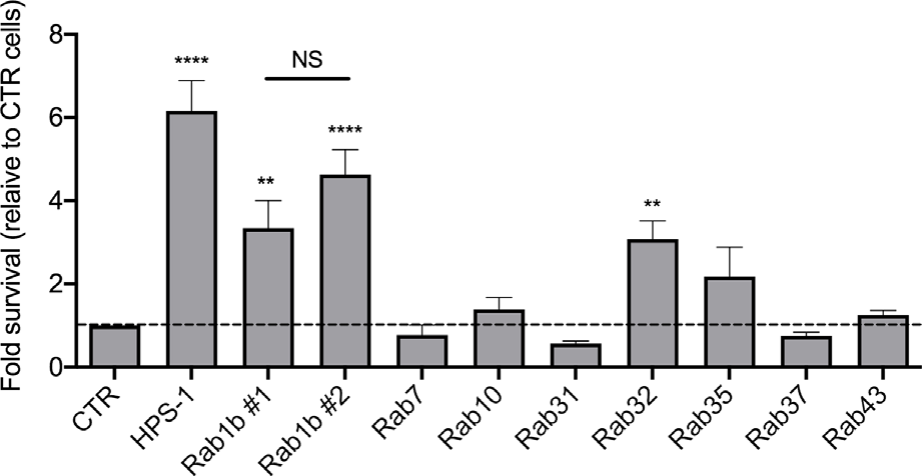
Validation of candidate genes. For each gene, lentiviral particles were generated and used to transduced iBMDMs. After selection with 5 µg/ml puromycin for 48 hours, cells were infected with *S*. Typhi*::mCherry*. At different times post-infection (1.5 and 24 hours), cells were lysed and CFUs enumerated. The CFUs obtained were normalized against 1.5 hpi and the data represent the fold-survival of the bacteria relative to in control (CTR) cells. Values are means ± SEM of at least two independent experiments performed in triplicates. *P* values were determined by the Student´s t test: **P < 0.01; ****P < 0,001. NS, non-significant.

For each candidate gene we took the second best shRNA (**Table S4**) and checked the levels of knockdown achieved (**Figure S4**). The reduction in mRNA expression was over 50% in all cases except for Rab27 and Rab40c. Furthermore, Rab25 mRNA was not detectable. This suggested that these three Rabs were false positives and were removed from our validation list. We then measured the CFUs 24 hours after infection with *S*. Typhi::*mCherry* (**Figure 4**). As expected, we confirmed that depletion of Rab32 and HPS-1 in mouse macrophages lead to an increased survival of *S*. Typhi (Spanò and Galán, 2012). Among the other 7 Rab GTPases tested, only the knockdown of Rab1b showed a significant increase of *S*. Typhi survival (**Figure 4**). It is extremely unlikely that two different shRNA sequences produce the same off-target phenotype; therefore, we tested the other Rab1b shRNA (Rab1b#1; z-score=1.78). The results obtained were similar to those reported above and are shown in **Figure 4**.

## Discussion

Unbiased genetic screenings have been crucial to identify host genes that play important roles in dealing with infections (Zhou et al., 2012; Li et al., 2017; Yeung et al., 2019). However, as primary macrophages undergo terminal differentiation and cannot be propagated in culture, the majority of these screens have been performed in alternative cell lines. Cell lines have the advantages of being easier to manipulate genetically and more straightforward to expand in culture, but their continuous subculture can lead to altered macrophage functions, including pathogen killing, limiting their suitability for screening experiments.

In this study, we performed a loss-of-function screen in primary mouse macrophages using viral-delivered shRNAs in the context of *S*. Typhi infection. To the best of our knowledge, this screen is the first of its kind. We have shown that viral-delivered shRNAs represent a good method to achieve sufficient level of knockdown for further phenotypic analyses. Importantly, we have developed a protocol that allows sorting and extracting good quality DNA from fixed cells for further next-generation sequencing. This is important due to the restrictions of working with *S*. Typhi, which in many places requires containment level 3 (CL3) facilities. Therefore, by fixing the samples we eliminated the requirement of a cell sorter in CL3 environment.

Our fluorescence microscopy and flow cytometry analyses of HPS-1-depleted macrophages in response to *S*. Typhi infection showed that the removal of HPS-1 led to a population of macrophages containing very high numbers of bacteria (>6), but also induced a shift in the intermediate population with more cells containing between 3 and 6 bacteria (**Figures 2C** and **S2**). Therefore, we selected a gate that includes both the very low abundant population containing a very high number of bacteria and the upper limit of the population containing lower numbers of intracellular bacteria (**Figure S3A**, gate H). This slightly more inclusive gating also allowed us to reduce the total number of cells to be sorted in order to recover enough cells from the H population for further analyses. The down side of this expansion is that we have probably increased the number of false positives. These aspects should be taken into consideration when planning for screens with larger libraries. Alternatives, such as performing a higher stringency secondary screen with libraries targeting the hits identified with a first low stringent strategy, should be carefully considered.

In addition to providing a proof-of-concept study, our screen revealed that Rab1b is important to control the growth of *S*. Typhi in mouse macrophages. Rab1 is known to regulate vesicle transport from the endoplasmic reticulum (ER) to the Golgi (Plutner et al., 1991). Moreover, Rab1 is involved in autophagy, a process by which the cell delivers cytosolic material (misfolded proteins, damaged organelles and pathogens) to the lysosome for degradation (Kakuta et al., 2017). In the context of bacterial infections, the active recruitment of Rab1 to vacuoles containing *Legionella pneumophila* or *Yersinia pestis*, prevents acidification of this compartment and permits their survival inside macrophages (Kagan et al., 2004; Connor et al., 2015). An siRNA screen in epithelial cells identified Rab1 as a host factor that facilitates *S*. Typhimurium intracellular growth (Thornbrough et al., 2012). Recent studies have identified three *S*. Typhimurium effectors neutralizing Rab1 functions: SseF and SseG block Rab1-mediated autophagy (Feng et al., 2018) while SseK3 covalently modifies Rab1 impairing ER-Golgi trafficking and limiting the secretion of cytokines (Gan et al., 2020; Meng et al., 2020). The fact that a pathogen uses multiple effectors to target a single host protein highlights the importance of Rab1 in the context of *Salmonella* infection. SseK3 is absent from *S*. Typhi while both SseG and SseF are present (including in the strain used in this study) and are 98.2% and 96.5% identical, respectively, to the *S*. Typhimurium effectors. However, SseF has evolved differently in different *Salmonella* serovars (Eswarappa et al., 2008) possibly explaining why *S*. Typhi cannot block Rab1b in mouse cells.

In summary, we have described experimental conditions to perform loss-of-function screens in primary macrophages negotiating the constraints required by a CL3 environment. This offers the potential to perform unbiased large-scale screenings in primary macrophages to identify novel mechanisms of pathogen killing. Interestingly, as a result of a targeted screen, we have identified a role of Rab1b in controlling the growth of *S*. Typhi in mouse macrophages. To the best of our knowledge, this is the first report showing a Rab GTPase other than Rab32 having a role in the restriction of *S*. Typhi in mice. Future work will define how Rab1b prevents *S*. Typhi growth in mouse macrophages and whether this strategy is related to other previously described macrophage-killing mechanisms against this human pathogen.

## Author´s Note

This paper is part of SS scientific legacy and this would have not been possible without her intelligence, vision and persistence. A dreadful destiny has snatched her from us too early, but her discoveries and ideas are living and flourishing.

## Data Availability Statement

The datasets presented in this study can be found in online repositories. The names of the repository/repositories and accession number(s) can be found below: https://www.ebi.ac.uk/ena, PRJEB43006.

## Author Contributions

VS-C performed the experiments and wrote the manuscript with input from MB and DC. RC performed experiments. VS-C, MB, and SS contributed to the design of the study and the analysis of the results. All authors contributed to the article and approved the submitted version.

## Funding

This work was supported by the European Union’s Horizon 2020 research and innovation program Marie Skłodowska-Curie Fellowship (706040_KILLINGTYPHI) to VS-C, the Wellcome Trust (Seed Award 109680/Z/15/Z), the European Union’s Horizon 2020 ERC consolidator award (2016-726152-TYPHI), the BBSRC (BB/N017854/1) and Tenovus Scotland (G14/19) to SS.

## Conflict of Interest

The authors declare that the research was conducted in the absence of any commercial or financial relationships that could be construed as a potential conflict of interest.
