# Supplementary material for: A Small-Scale shRNA Screen in Primary Mouse Macrophages Identifies a Role for the Rab GTPase Rab1b in Controlling Salmonella Typhi Growth
Source: Front Cell Infect Microbiol. 2021 Apr 7;11:660689. doi: 10.3389/fcimb.2021.660689 (PMC8059790; doi:10.3389/fcimb.2021.660689)
Supplement: Supplementary file 1 [file DataSheet_1.docx]

**
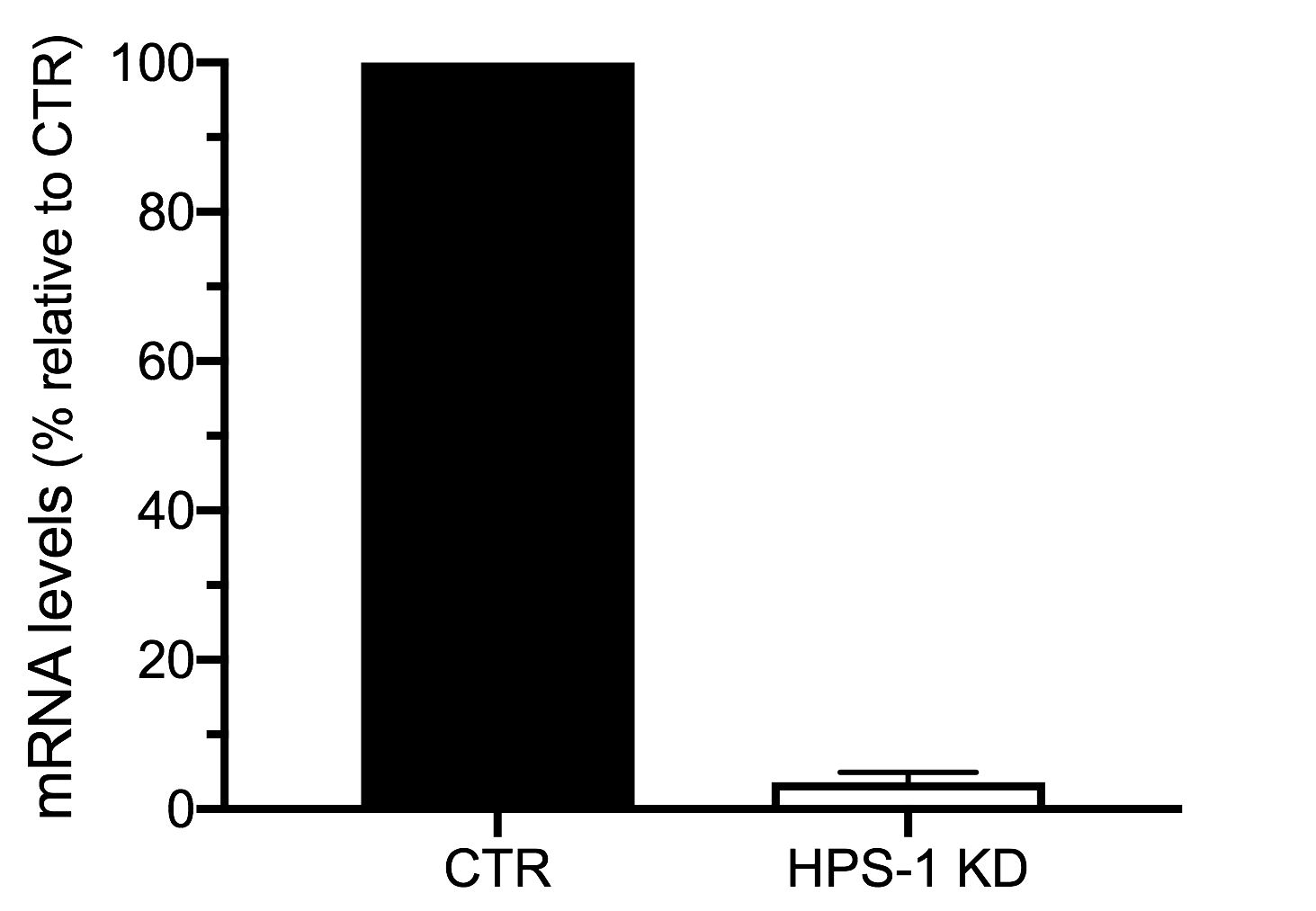
Supplementary Figure 1.** **mRNA levels of HPS-1 in BMDMs.** BMDMs were transduced with an shRNA targeting HPS-1 (HPS-1 KD) or non-targeting sequences (CTR) and the transcript levels of HPS-1 were determined by RT-qPCR. The GAPDH gene was used as reference.

**Supplementary
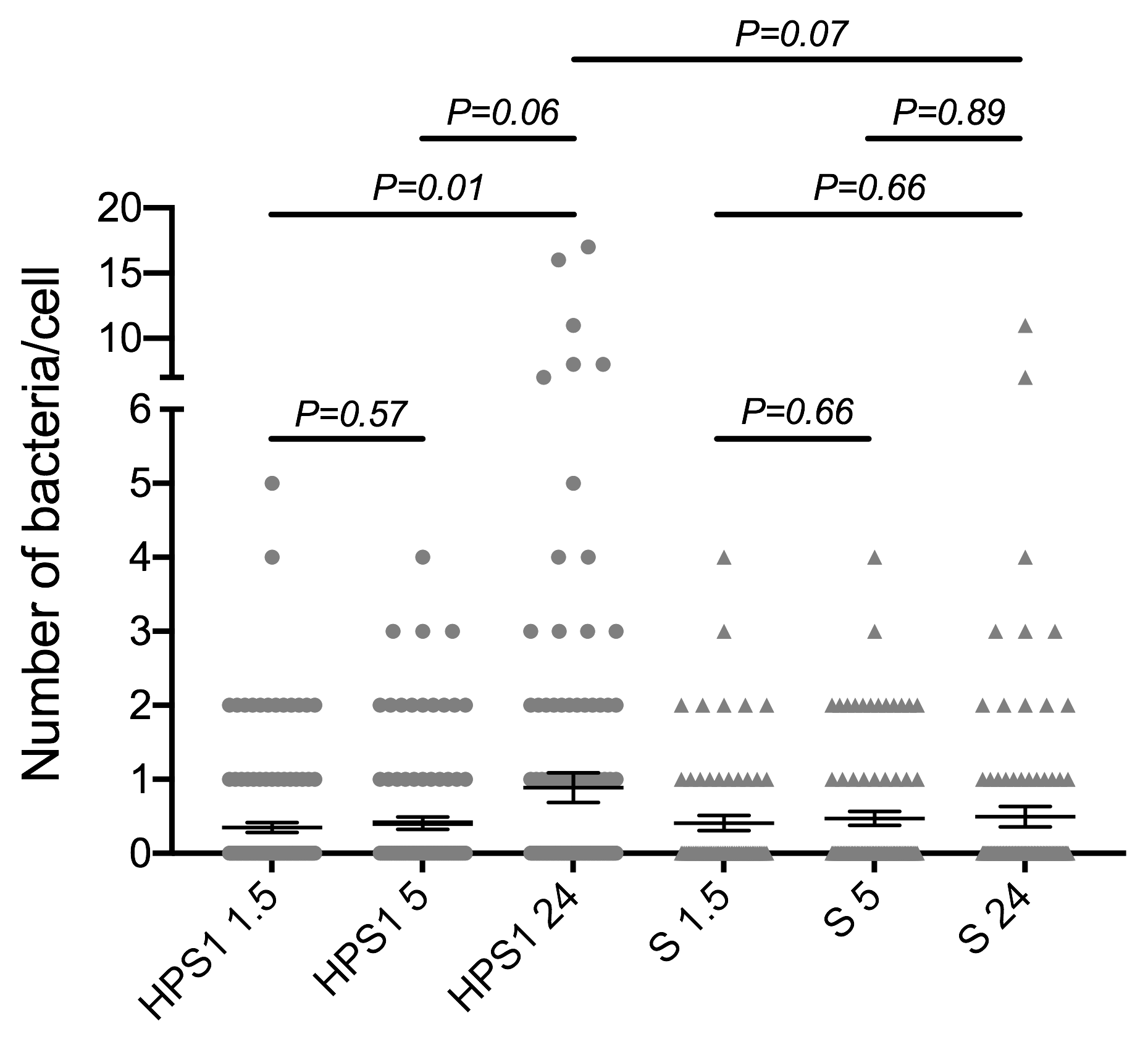
Figure 2**. **shRNA-mediated knockdown in BMDMs.** BMDMs HPS-1 Knockdown (HPS1) or control (Scrambled shRNA; S) were infected with *S*. Typhi::*mCherry* (MOI 10). At different times post-infection, cells were fixed with 4% PFA and analyzed by fluorescence microscopy to determine the number of bacteria per cell. Student´s t-test (unpaired, two tailed) was used to calculate statistical significance of the data and the *P* values obtained are indicated.

**Supplementary
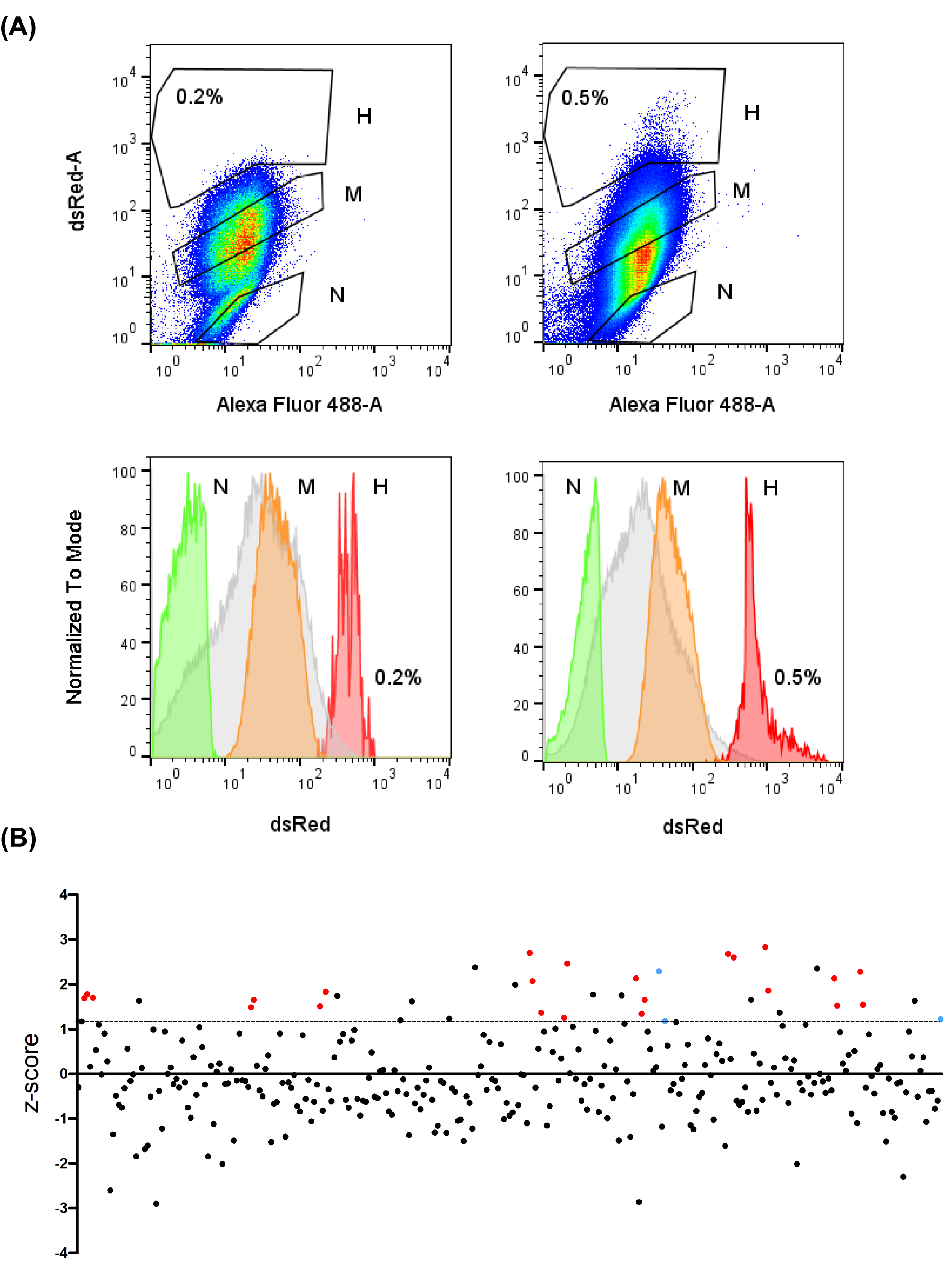
Figure 3**. **Cell sorting by flow cytometry and analysis of the results (z-score) (A)** Samples ar 1.5 and 24 hours-post infection were analyzed and sorted in a BD Influx BSLII Sorter (Ian Fraser Cytometry Centre, University of Aberdeen). For a more accurate separation of the different cellular subpopulations, cell auto-fluorescence (Alexa Fluor 488-A) was used together with bacteria fluorescence (dsRed) to select sorting gates. Sorting gates used to isolate the different cell sub-populations are indicated as well as the percentage of cells carrying high amount of intracellular bacteria. The subpopulations sorted are shown based in dsRed fluorescence in the lower panel. N (non-infected), M (infected cells with low number of intracellular bacteria), H (infected cells with high number of intracellular bacteria) **(B)** Dot-plot of z-scores. Genes exhibiting more than 1 hairpin with a z-score >1.18 are represented in red. Rab32 and HPS-1 (positive controls) are represented in blue.

**Supplementary
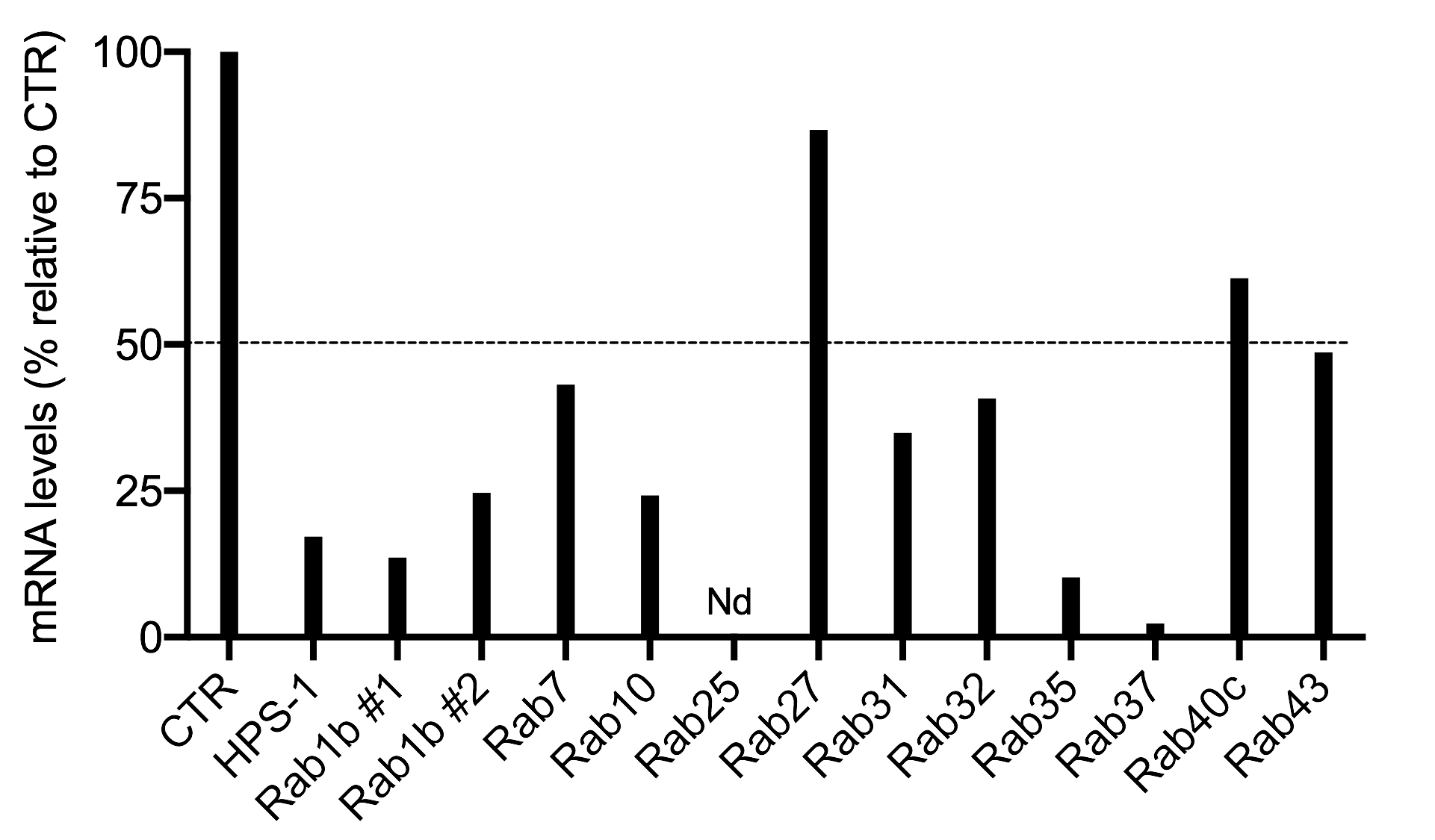
Figure 4. mRNA levels of Rab GTPases validated**. iBMDMs were transduced with the second best shRNA of each gene or non-targeting sequences (CTR) and the transcript levels of each gene were determined by RT-qPCR. The GAPDH gene was used as reference.
