# Supplementary material for: A Small-Scale shRNA Screen in Primary Mouse Macrophages Identifies a Role for the Rab GTPase Rab1b in Controlling Salmonella Typhi Growth
Source: Front Cell Infect Microbiol. 2021 Apr 7;11:660689. doi: 10.3389/fcimb.2021.660689 (PMC8059790; doi:10.3389/fcimb.2021.660689)
Supplement: Supplementary file 2 [file Table_1.docx]

**Table S1.** shRNA library comprising the mouse Rab GTPases and HPS-1 (positive control)

| **GENE** | **TRCN_ID** | **shRNA SEQUENCE (SENSE STRAND)** |
| --- | --- | --- |
| Rab1b | TRCN0000100822 | GATTTCAAGATTCGAACCATT |
| Rab1b | TRCN0000100823 | CATGGCATCATTGTGGTGTAT |
| Rab1b | TRCN0000302710 | CCACGCACCTTTCTTTGGAAT |
| Rab1b | TRCN0000302711 | GCCAAGAATGCCACCAATGTT |
| Rab1b | TRCN0000302786 | GCGGTTTGCTGATGACACTTA |
| Rab1b | TRCN0000379948 | ACACCACGGCCAAGGAATTTG |
| Rab1b | TRCN0000381762 | AGTCCTACGCCAACGTGAAAC |
| Rab2b | TRCN0000100406 | ACGGCCTTATATTCATGGAAA |
| Rab2b | TRCN0000100408 | AGGTTTATTTGATGTCCACAA |
| Rab2b | TRCN0000287555 | GCAGTCAATTACCTCCTCAGT |
| Rab2b | TRCN0000287556 | CCTCCTTCAGTTTACCGACAA |
| Rab2b | TRCN0000287557 | CCGCGTAGATTATGGCTCTTT |
| Rab3a | TRCN0000089148 | GCCTTATACTTTGGGATAAAT |
| Rab3a | TRCN0000089149 | CGACTATATGTTCAAGATCCT |
| Rab3a | TRCN0000089150 | ACCAATGAGGAGTCATTTAAT |
| Rab3a | TRCN0000089151 | TCACCAATGAGGAGTCATTTA |
| Rab3a | TRCN0000089152 | CAGCGCCAAGGACAACATTAA |
| Rab3b | TRCN0000089363 | GCAGTCTTTGATAAACTGTAA |
| Rab3b | TRCN0000089364 | GCAGCAGAACTGCTCTTGTTA |
| Rab3b | TRCN0000089366 | CGATAAGATGTCTGACTCGAT |
| Rab3b | TRCN0000089367 | CTTCAAAGTGAAGACAGTCTA |
| Rab3c | TRCN0000089453 | CCTGTGTTAATATGTGGCAAA |
| Rab3c | TRCN0000089454 | CGATTCCTTTACATCTGCATT |
| Rab3c | TRCN0000089455 | GATAACATCAACGTGAAGCAA |
| Rab3c | TRCN0000089456 | CCCAGGTTATCCTGGCTGGAA |
| Rab3c | TRCN0000089457 | TGTTCCGTTATGCCGACGATT |
| Rab3d | TRCN0000089443 | CGGTGCAAATTATGTTCTTAA |
| Rab3d | TRCN0000089444 | CAAACTGCTCTTGATCGGGAA |
| Rab3d | TRCN0000089445 | CATCATCTGTGACAAGATGAA |
| Rab3d | TRCN0000089446 | CTACCGACATGACAAGAGGAT |
| Rab3d | TRCN0000089447 | CCGACATGACAAGAGGATCAA |
| Rab4a | TRCN0000088973 | GCTTTCTCATTGCGTTGGTTA |
| Rab4a | TRCN0000088974 | GATAATAAATGTCGGTGGTAA |
| Rab4a | TRCN0000088975 | AGATGACTCAAATCATACCAT |
| Rab4a | TRCN0000088976 | CCTACAATGCGCTTACTAATT |
| Rab4a | TRCN0000088977 | CTCAAATCATACCATAGGAAT |
| Rab4b | TRCN0000089433 | CCACCAGTTTATTGAGAATAA |
| Rab4b | TRCN0000089434 | GCATTCAGTATGGCGACATAT |
| Rab4b | TRCN0000089435 | TCCACCAGTTTATTGAGAATA |
| Rab4b | TRCN0000089436 | AGACTGTGAAACTACAGATTT |
| Rab4b | TRCN0000089437 | CATACAACTCACTCGCTGCTT |
| Rab5a | TRCN0000100795 | CCCAAGCAAATGGTGTAATTT |
| Rab5a | TRCN0000100796 | GCAGCCATAGTTGTGTATGAT |
| Rab5a | TRCN0000100797 | CGCTTTGTGAAAGGCCAATTT |
| Rab5a | TRCN0000100798 | GCTGGTCAAGAACGGTATCAT |
| Rab5a | TRCN0000100799 | CAAGCAGCCATAGTTGTGTAT |
| Rab5b | TRCN0000100620 | GCACTTTAATTGATGGTAGTT |
| Rab5b | TRCN0000100621 | CCGTGTGTTTAGATGACACAA |
| Rab5b | TRCN0000100622 | CCTGGCAATAGCAAAGAAGTT |
| Rab5b | TRCN0000100623 | GCAGATGACAACAGCTTATTA |
| Rab5b | TRCN0000100624 | CGTGGTCTATGATATTACTAA |
| Rab5c | TRCN0000100745 | CCCGACTGGAATCTACTCTAA |
| Rab5c | TRCN0000100746 | CCCAACATCGTCATTGCACTA |
| Rab5c | TRCN0000100747 | GCAATGAACGTGAATGAAATT |
| Rab5c | TRCN0000100748 | GCAACAAGATCTGTCAGTTTA |
| Rab5c | TRCN0000100749 | GCTAAGAAGCTTCCCAAGAAT |
| Rab6b | TRCN0000100900 | CCACAGTCAAATCCAACTTTA |
| Rab6b | TRCN0000100901 | CGGGATTGACTTCTTGTCAAA |
| Rab6b | TRCN0000100902 | CCAGCAGACTTCTAAATGGAT |
| Rab6b | TRCN0000100903 | CTGTGGTGGTATATGACATTA |
| Rab6b | TRCN0000100904 | GCTGTGGTGGTATATGACATT |
| Rab7 | TRCN0000100880 | GCGGCAGTATTCTGTACAGTA |
| Rab7 | TRCN0000100881 | TGAACCCATCAAACTGGACAA |
| Rab7 | TRCN0000100882 | GAAGTTCAGTAACCAGTACAA |
| Rab7 | TRCN0000100883 | TGCTGTGTTCTGGTGTTTGAT |
| Rab7 | TRCN0000100884 | GCCCTTAAACAGGAAACAGAA |
| Rab8a | TRCN0000100420 | CCATGAAATGAATCTGTCTTT |
| Rab8a | TRCN0000100421 | CGCCTTCAACTCCACATTCAT |
| Rab8a | TRCN0000100422 | CGGAATTGGATTCGGAACATT |
| Rab8a | TRCN0000100423 | CTACGACATTACCAATGAGAA |
| Rab8a | TRCN0000100424 | CTCGATGGCAAGAGGATTAAA |
| Rab8b | TRCN0000100535 | GCCAAGAACTAACAGAACTTT |
| Rab8b | TRCN0000100536 | CCGAACAATTACGACAGCATA |
| Rab8b | TRCN0000100537 | CCGGTCTAAGAAGACCAGTTT |
| Rab8b | TRCN0000100538 | CAGGAAAGATTCCGAACAATT |
| Rab8b | TRCN0000100539 | CAAATGTGATATGAACGACAA |
| Rab8b | TRCN0000295363 | AGTGCAAAGTCGAGTACAAAT |
| Rab8b | TRCN0000295424 | TTTACACTTGCACGGGATATA |
| Rab8b | TRCN0000295425 | AGAAGTTAGCAATTGACTATG |
| Rab8b | TRCN0000380343 | ATAACAGAGAGCCGGTCTAAG |
| Rab9b | TRCN0000100660 | GCCAAGTAGTTTATGTCATTT |
| Rab9b | TRCN0000100661 | CCGTTATGTAACCAACAAGTT |
| Rab9b | TRCN0000100662 | GCAGATGTAAAGGACCCAGAT |
| Rab9b | TRCN0000100663 | GAGCCTTAGAACACCATTCTA |
| Rab9b | TRCN0000100664 | GTAGAGGAACAGCTGGAACAT |
| Rab10 | TRCN0000100835 | GCCTATTAACTGTCAGTTAAT |
| Rab10 | TRCN0000100839 | CATGCCAATGAAGATGTGGAA |
| Rab10 | TRCN0000335543 | CGATGCCTTCAATACCACCTT |
| Rab10 | TRCN0000335544 | GAGAGTTGTACCGAAAGGCAA |
| Rab10 | TRCN0000335623 | GCGTTCCTCACATTAGCTGAA |
| Rab10 | TRCN0000348573 | GACTGCTTGCGGACTATTATA |
| Rab11a | TRCN0000100340 | CCCTGTAAACATAACAGCATT |
| Rab11a | TRCN0000100342 | CAGGGCTATAACGTCTGCATA |
| Rab11a | TRCN0000100343 | ACCTCTTTAAAGTTGTCCTTA |
| Rab11a | TRCN0000100344 | CAGAGATATACCGCATTGTTT |
| Rab11a | TRCN0000305795 | AGTAGGTGCCTTATTGGTTTA |
| Rab11a | TRCN0000305864 | TAACCTCCTGTCTCGATTTAC |
| Rab11a | TRCN0000324936 | GAGAGATCATGCTGATAGTAA |
| Rab11b | TRCN0000100255 | CCTGTCTGCAAGTGAAGCAAT |
| Rab11b | TRCN0000100256 | CCTCACAGAAATCTACCGTAT |
| Rab11b | TRCN0000100257 | CCTATTCAAAGTGGTGCTTAT |
| Rab11b | TRCN0000100258 | CAAGACCATCAAGGCTCAGAT |
| Rab11b | TRCN0000100259 | CCTAGAGAGCAAGAGTACCAT |
| Rab13 | TRCN0000100855 | GAGCATTTCTTGCCTCCTATT |
| Rab13 | TRCN0000100856 | GCCAAGAACGATTCAAGACAA |
| Rab13 | TRCN0000100857 | CCAAGAACGATTCAAGACAAT |
| Rab13 | TRCN0000100858 | CGAGAGCACAGAATCCGATTT |
| Rab13 | TRCN0000100859 | GATGAGAAATCCTTCGAGAAT |
| Rab14 | TRCN0000089374 | GCACAGAGAGATGTTACCTAT |
| Rab14 | TRCN0000089376 | CCAATCCAAACACTGTAATAA |
| Rab14 | TRCN0000306372 | ATCACCAGAAGAAGTACATAT |
| Rab14 | TRCN0000306430 | CGGTTACACGGAGCTACTATA |
| Rab14 | TRCN0000332297 | ACCAATCCAAACACTGTAATA |
| Rab14 | TRCN0000332372 | CAGAGAGATGTTACCTATGAA |
| Rab14 | TRCN0000332373 | CCTTTGATTGTCCTTGTGATA |
| Rab15 | TRCN0000093169 | CCCTGAAGTATAGCAACAGAA |
| Rab15 | TRCN0000093170 | CCAGACTATCACAAAGCAGTA |
| Rab15 | TRCN0000093171 | GCATGGACTTCTACGAAACAA |
| Rab15 | TRCN0000093172 | CGGTGTTGACTTTAAGATGAA |
| Rab15 | TRCN0000093173 | GCGCTCCTATCAGCATATCAT |
| Rab17 | TRCN0000100915 | GCTGCCTCTTTGTCCATTCAT |
| Rab17 | TRCN0000100916 | CCGGTACATGAAGCAGGACTT |
| Rab17 | TRCN0000100917 | TCTGAGATCTTCAACACTGTT |
| Rab17 | TRCN0000100918 | CTCCTGGTTTATGACATCACT |
| Rab17 | TRCN0000100919 | GCGCCAGTGCTGTGCACGATA |
| Rab18 | TRCN0000177028 | GATGGAAATAAGGCTAAACTT |
| Rab18 | TRCN0000177794 | CGATAGAAATGAAGGCTTGAA |
| Rab18 | TRCN0000177937 | GCAACAATAGGTGTTGACTTT |
| Rab18 | TRCN0000178291 | GCTAAACTTGCAATATGGGAT |
| Rab18 | TRCN0000181812 | GAACCAGAACAAAGGAGTCAA |
| Rab18 | TRCN0000182111 | CCCAGCTATTATAGAGGTGCA |
| Rab18 | TRCN0000197499 | CAGGGAGTTATATTAGTCTAT |
| Rab18 | TRCN0000215331 | CAAGAGAGGTTCAGAACATTA |
| Rab18 | TRCN0000215879 | CTTGTTGAGAAGATCATTCAG |
| Rab18 | TRCN0000216622 | GTAAACATGCTAGTTGGAAAT |
| Rab20 | TRCN0000102640 | CCTGACAGAAACAGCCAACAA |
| Rab20 | TRCN0000102642 | CCTCCTCTTTGAAACCTTGTT |
| Rab20 | TRCN0000102643 | CCCTTTACAAGAAGATCCTGA |
| Rab20 | TRCN0000102644 | GAAGATCCTGAAGTACAAGAT |
| Rab21 | TRCN0000102680 | CCTACTTAAAGGCTTCATTTA |
| Rab21 | TRCN0000102681 | GCCCAATTTACTACCGAGATT |
| Rab21 | TRCN0000102682 | GCATTACCATACTTCCGCTAA |
| Rab21 | TRCN0000102683 | GCAGGCATCTTTCTTAACAAA |
| Rab21 | TRCN0000102684 | GCTAAACAGAACAAAGGCATT |
| Rab22a | TRCN0000100830 | GCCGGTGTGTATTGATCGTTT |
| Rab22a | TRCN0000100833 | GCTGGACAAGAACGATTTCGT |
| Rab22a | TRCN0000100834 | CGCAGATTCCATTCATGCCAT |
| Rab22a | TRCN0000302790 | GCAGGGAACAAGTGCGATCTT |
| Rab22a | TRCN0000302791 | CCGAATATCAATCCAACCATA |
| Rab22a | TRCN0000376917 | ATGGATGGTAGGATTGAATTG |
| Rab22a | TRCN0000380076 | GACGCCACCTCATGCTCTTTA |
| Rab22a | TRCN0000381994 | TGTCAGAGTCGTATCAGTAAG |
| Rab23 | TRCN0000102770 | GCCAAACACTACCGCTAATTT |
| Rab23 | TRCN0000102771 | GCTGGATGATTCATGCATCAA |
| Rab23 | TRCN0000102772 | CGACAGATTCAGGTTAACGAT |
| Rab23 | TRCN0000102773 | ACACTCAAGTAGTAACAAGAT |
| Rab23 | TRCN0000102774 | CATCAACCTTAGACCTAACAA |
| Rab24 | TRCN0000102805 | CCCAGTGGAATTAGATGAATT |
| Rab24 | TRCN0000102806 | CGTCGTGTAGACTTCCATGAT |
| Rab24 | TRCN0000102807 | GCCGATAATATCAAAGCCCAA |
| Rab24 | TRCN0000102809 | AGCCATGAGCAGAATCTATTA |
| Rab24 | TRCN0000306308 | ATCTACCTGTGTGGCACTAAG |
| Rab24 | TRCN0000326454 | GAGAGCCAAGTTCTGGGTTAA |
| Rab24 | TRCN0000382512 | TCAGGACTATGCCGATAATAT |
| Rab25 | TRCN0000100250 | CCTGCCTTCAGCTTTCAGATA |
| Rab25 | TRCN0000100251 | CCTGGTATTTGACCTGACCAA |
| Rab25 | TRCN0000100252 | GCCCTCGACTCCACCAATGTT |
| Rab25 | TRCN0000100253 | CTTTGTCTTTAAGGTGGTGCT |
| Rab25 | TRCN0000100254 | CCTCAAAGAGATCTTTGCAAA |
| Rab25 | TRCN0000238206 | ATATCTCCACCTCCCTTACTG |
| Rab25 | TRCN0000238207 | GCTGGCTAAAGGAGCTGTATG |
| Rab25 | TRCN0000238208 | CATGCCGAAGCCACGATTGTT |
| Rab26 | TRCN0000341409 | TGAGAATTGCCCGAGTCTATA |
| Rab26 | TRCN0000341411 | CAGGCTGCATGACTATGTTAA |
| Rab26 | TRCN0000341412 | TCTACGACATCACCAACAAAG |
| Rab26 | TRCN0000341413 | ACTCAAGACCGTGTGGTAAAG |
| Rab26 | TRCN0000341481 | GGCATCGACTTCCGGAATAAA |
| Rab27a | TRCN0000100575 | GCCAGTTTAAGAGAAGTGTTT |
| Rab27a | TRCN0000100576 | CGAAACTGGATAAGCCAGCTA |
| Rab27a | TRCN0000100577 | GCTTCTGTTCGACCTGACAAA |
| Rab27a | TRCN0000100578 | CCAGTACACTGATGGCAAGTT |
| Rab27a | TRCN0000100579 | GACAAACATAAGCCACGCGAT |
| Rab27b | TRCN0000100425 | CCTGAGACAATGTCAAACCAT |
| Rab27b | TRCN0000100426 | CGGGAAGACAACATTTCTCTA |
| Rab27b | TRCN0000100427 | GCATACCATACTTCGAAACAA |
| Rab27b | TRCN0000100428 | GCTTCTGGACTTAATCATGAA |
| Rab27b | TRCN0000100429 | CTCTATAGATACACAGACAAT |
| Rab28 | TRCN0000100695 | GCTTCAAATGAGGCTGTAATA |
| Rab28 | TRCN0000100696 | CCTGGGAATCAAATTAAACAA |
| Rab28 | TRCN0000100697 | GCAGACGATAGGACTGGATTT |
| Rab28 | TRCN0000100698 | GCACACACTGTATTGATAGTT |
| Rab28 | TRCN0000100699 | GCTGATAAGCACTTACGATTT |
| Rab7l1 | TRCN0000102730 | CTCTGAGTCATTCCAATTCTT |
| Rab7l1 | TRCN0000102731 | CCATGACACGACTCTACTATA |
| Rab7l1 | TRCN0000102732 | GTTCAGTAAAGAGAATGGCTT |
| Rab7l1 | TRCN0000102733 | GACTCTACTATAGAGATGCTT |
| Rab7l1 | TRCN0000102734 | CAATGCCACTACTTTCAGCAA |
| Rab30 | TRCN0000100401 | CAGCTATTTGACTTGTTGTAA |
| Rab30 | TRCN0000100402 | CGAAGATTCACTCAGGGTCTT |
| Rab30 | TRCN0000348722 | ACGTGCCTAGTCCGAAGATTC |
| Rab30 | TRCN0000351883 | CGGGAGATAGAACAGTATGCT |
| Rab30 | TRCN0000351884 | GCAGTCTTCTTCAGTCTCATT |
| Rab30 | TRCN0000351959 | CTTGATCCTTACCTATGACAT |
| Rab31 | TRCN0000100435 | GCGGGTTTGTATATGTGTAAA |
| Rab31 | TRCN0000100436 | GCAGGATTCATTTCATACCTT |
| Rab31 | TRCN0000100437 | CCAGGATCACTTTGACCACAA |
| Rab31 | TRCN0000100438 | CAGCGCGAAGAATGCCATTAA |
| Rab31 | TRCN0000100439 | CGCGAAGAATGCCATTAACAT |
| Rab31 | TRCN0000379445 | ACCGTGCCTTGTGGAAATGAA |
| Rab31 | TRCN0000380370 | GGGTTGGGAAATCCAGCATTG |
| Rab31 | TRCN0000380682 | CGCTAAGGAGTACGCTGAATC |
| Rab31 | TRCN0000381460 | TCAAGGAATCAGCCGCCAGAT |
| Rab31 | TRCN0000381947 | TGGCGATTGCTGGGAACAAGT |
| Rab32 | TRCN0000102686 | CTACATTTGATGCAGTCCTAA |
| Rab32 | TRCN0000102687 | CCTCTGCCAAGGATAATATAA |
| Rab32 | TRCN0000102688 | CGTGGGTAAGACGAGCATCAT |
| Rab32 | TRCN0000102689 | GAAATCGACCTGGACAGAATT |
| Rab32 | TRCN0000288450 | GCCAAGTTTCTGTAGTGTAAA |
| Rab33a | TRCN0000100725 | GCTCATAACATGCTCTTGTTT |
| Rab33a | TRCN0000100726 | GACCTCCTTCACCAACTTAAA |
| Rab33a | TRCN0000100727 | CTCCAACTTAGCCCTGAAATT |
| Rab33a | TRCN0000100728 | CAGTACGTGCAGATTCGCATT |
| Rab33b | TRCN0000100600 | GCCCAATACATCTTTCTTAAT |
| Rab33b | TRCN0000100601 | GCGAACGACATACCTCGAATT |
| Rab33b | TRCN0000100602 | GATAACAGAATTAGCCTGAAA |
| Rab33b | TRCN0000100603 | GCTTGGATAGAGGAATGCAAA |
| Rab33b | TRCN0000100604 | CCACGTAGAAGCTATATTCAT |
| Rab34 | TRCN0000100520 | CCTGGACATTTGCACTGACTT |
| Rab34 | TRCN0000100521 | CCAAGAGATTAAGGCCGAGTA |
| Rab34 | TRCN0000100522 | GCACTAACCTTTGAGGCCAAT |
| Rab34 | TRCN0000100523 | CGTGGGATTTAAGATATCCAA |
| Rab34 | TRCN0000302686 | CGGCACATTGCAGATGTTGTT |
| Rab34 | TRCN0000304930 | CTGCAAAGACACCTTCGATAA |
| Rab34 | TRCN0000304932 | TGAGTACTCCTGCTCAGTATT |
| Rab34 | TRCN0000311168 | GGCTACCATCGGAGTGGATTT |
| Rab34 | TRCN0000374432 | ATCGTTGTGGGAGACCTATCT |
| Rab34 | TRCN0000380869 | GACAGGCACCGTGGGATTTAA |
| Rab35 | TRCN0000100530 | CGTAACTCAGAAGAACTGATT |
| Rab35 | TRCN0000100531 | GCCGAATATTAGTGGGCAATA |
| Rab35 | TRCN0000100532 | GCTGTTACGATTCGCAGACAA |
| Rab35 | TRCN0000100533 | CGAGTCCTTTGTCAACGTCAA |
| Rab35 | TRCN0000100534 | AGTGCCAAGGAGAACGTCAAT |
| Rab35 | TRCN0000379544 | CTCAGTTTAGTGCCGTTATTT |
| Rab35 | TRCN0000379586 | CAAGCGATGGCTTCATGAAAT |
| Rab35 | TRCN0000379707 | ACCATCACCTCTACGTATTAT |
| Rab35 | TRCN0000380231 | GACAGAAGATGCCTACAAATT |
| Rab36 | TRCN0000100800 | CCCATCTTTCTCCTGCCATAA |
| Rab36 | TRCN0000100801 | CCATGATTACAAGGCCACGAT |
| Rab36 | TRCN0000100802 | CCTCATTCACAGGTTGTGCAA |
| Rab36 | TRCN0000100803 | GATGGAGACCTAATACGAATA |
| Rab36 | TRCN0000100804 | CAAGTGTATTGCGTCTGCCTA |
| Rab37 | TRCN0000100815 | GCAGAACTGAACAAAGCCATA |
| Rab37 | TRCN0000100816 | CCAGGGAATATGGTGTTCCTT |
| Rab37 | TRCN0000100817 | GACGTGGTGATTATGCTTCTA |
| Rab37 | TRCN0000100818 | GCCCAGAGAGACGTGGTGATT |
| Rab37 | TRCN0000100819 | CGGCATAGACTCCAGGAATAA |
| Rab38 | TRCN0000102645 | GCCCTAATATTTGTTCCTTTA |
| Rab38 | TRCN0000102646 | GCTTCGTAGGATGGTTTGAAA |
| Rab38 | TRCN0000102647 | GAGTCTATAGAACCGGACATT |
| Rab38 | TRCN0000102648 | ACCAGCATTATCAAGCGCTAT |
| Rab38 | TRCN0000102649 | CACATTTGAAGCCGTGGCAAA |
| Rab39 | TRCN0000102695 | CCGTCTTAGAAATACTGACTA |
| Rab39 | TRCN0000102696 | CGCAACTCAGTTGGAGGATTT |
| Rab39 | TRCN0000102697 | GCTTCAGATCAATAACTCGAT |
| Rab39 | TRCN0000102698 | AGATCAATAACTCGATCCTAT |
| Rab39 | TRCN0000102699 | GACTGTGGAATGAAGTACATA |
| Rab39b | TRCN0000102745 | CCCAGGATTATCCAGTGGATA |
| Rab39b | TRCN0000102746 | CCCTACCAAATTGTATTTGTT |
| Rab39b | TRCN0000102747 | CGGCTCATTGTCATCGGCGAT |
| Rab39b | TRCN0000102748 | CGCTTTGCTCAGGTTTCAGAT |
| Rab39b | TRCN0000102749 | GCCTACTACAGGAATTCAGTA |
| Rab40b | TRCN0000102765 | GCATTTATTAACGGTAACGAT |
| Rab40b | TRCN0000102766 | CGATGGATTAAGGAGATTGAT |
| Rab40b | TRCN0000102767 | CCACCTTAAGTCTTTCTCGAT |
| Rab40b | TRCN0000102768 | CTTCAACATTACAGAGTCCTT |
| Rab40b | TRCN0000102769 | GTGGTCCTGGTCTATGACATT |
| Rab40c | TRCN0000054849 | GCCGTGCCATTGTCTCCTGTA |
| Rab40c | TRCN0000054850 | CTGTACCATCTTCAGGTCCTA |
| Rab40c | TRCN0000054851 | GCAACAGCCTTAAGAGGTCTA |
| Rab40c | TRCN0000054852 | CCACTACCTGTCACCATCAAA |
| Rab40c | TRCN0000287392 | CTGTGCAACTTCAACGTCATT |
| Rab40c | TRCN0000294887 | AGCAACGGGATAGACTATAAG |
| Rab40c | TRCN0000307449 | AGTGTTGAAGCCAGATCTTTA |
| Rab40c | TRCN0000380513 | TGGATCAAGGAGATCGATGAG |
| Rab42 | TRCN0000341460 | ACGTCACTGCTACGGTGTTAC |
| Rab42 | TRCN0000341464 | CTGACAAGGTGGTCTTCTTAC |
| Rab42 | TRCN0000341466 | TGACGAACAGAGAGTCCTTTG |
| Rab42 | TRCN0000341467 | TGGGCGTTCTGTTGGTCTTTG |
| Rab42 | TRCN0000341469 | ACCTGAACACCCGATGCGTAT |
| Rab43 | TRCN0000102811 | AGGTCCCATGTTCAGTGAGAA |
| Rab43 | TRCN0000318248 | ACAAGTCAGACCTTGCCGATT |
| Rab43 | TRCN0000318249 | GAGCACTATGACATCCTCTGT |
| Rab43 | TRCN0000318322 | GATCGAGGATGTGAGGAAGTA |
| Rab43 | TRCN0000318323 | CCTGCCTCTTACCAAAGTATA |
| Rabl2a | TRCN0000100885 | CCCTACATTTCTGGAGAACAT |
| Rabl2a | TRCN0000100886 | GCAGACATACAGATGACTCAA |
| Rabl2a | TRCN0000100887 | CCCAAGACACAGACCCATATT |
| Rabl2a | TRCN0000100888 | GCACCACACATTTCCTTCTTT |
| Rabl2a | TRCN0000100889 | GTCCAAACTCATGGAGAGATT |
| Rabl3 | TRCN0000181097 | CCCAGAGTAACGAACTCATAT |
| Rabl3 | TRCN0000182964 | CACAATCAAGTGTTAGGAAAT |
| Rabl3 | TRCN0000241041 | TGAAGAGAAGACATACTATAT |
| Rabl3 | TRCN0000241042 | TTGCTGCTGAAGGCTTAATTA |
| Rabl4 | TRCN0000102720 | ACTGTCCTGAAGGGCTGCCCA |
| Rabl4 | TRCN0000102721 | GCCTGGAATTCTTTGAGACAT |
| Rabl4 | TRCN0000102722 | CCTTGTCTATGATGTGACCAA |
| Rabl4 | TRCN0000102723 | CTGGATAAGTTGTGGGAGAAT |
| Rabl4 | TRCN0000102724 | CAGTGCCAGTTCTTGACACAA |
| Rabl5 | TRCN0000191434 | CCACCTAAAGGAAATTGAAAT |
| Rabl5 | TRCN0000191988 | GTGGAGTTCATCAAGTATTTA |
| Rabl5 | TRCN0000200816 | GCCACCTAAAGGAAATTGAAA |
| Rabl5 | TRCN0000215745 | GATCGTCTTCAATGCTGATAT |
| Rabl5 | TRCN0000247292 | GGTGGAGTTCATCAAGTATTT |
| Rabl5 | TRCN0000247293 | TCCCAAAGGACGCTGACTAAC |
| Rabl5 | TRCN0000257636 | TCGTCTTCAATGCTGATATTC |
| Rabl5 | TRCN0000257657 | CTGAAGCTGGTGCACTCAAAC |
| Rabl5 | TRCN0000257762 | CTTCGGACATCACTGAATATA |
| Hps-1 | TRCN0000292556 | GCAAGCTGTTGGCTTTCTACT |
