# Supplementary material for: A Small-Scale shRNA Screen in Primary Mouse Macrophages Identifies a Role for the Rab GTPase Rab1b in Controlling Salmonella Typhi Growth
Source: Front Cell Infect Microbiol. 2021 Apr 7;11:660689. doi: 10.3389/fcimb.2021.660689 (PMC8059790; doi:10.3389/fcimb.2021.660689)
Supplement: Supplementary file 5 [file Table_4.docx]

**Table S4.** Candidate genes identified from the screen and shRNA for validation

**(A)** List of genes with a z-score >1.18

| **Z-score (>1.18)** | **Gene name** | **TRCN** |
| --- | --- | --- |
|  |  |  |
| 2.83 | Rab37 | TRCN0000100817 |
| 2.70 | Rab25 | TRCN0000100252 |
| 2.68 | Rab35 | TRCN0000379586 |
| 2.60 | Rab35 | TRCN0000100532 |
| 2.46 | Rab27a | TRCN0000100577 |
| 2.38 | Rab22a | TRCN0000302790 |
| 2.35 | Rab40b | TRCN0000102769 |
| 2.29 | Rab32 | TRCN0000102688 |
| 2.28 | Rab43 | TRCN0000318248 |
| 2.13 | Rab40c | TRCN0000380513 |
| 2.13 | Rab31 | TRCN0000381460 |
| 2.07 | Rab25 | TRCN0000238207 |
| 1.99 | Rab24 | TRCN0000306308 |
| 1.86 | Rab37 | TRCN0000100818 |
| 1.83 | Rab10 | TRCN0000335543 |
| 1.78 | Rab1b | TRCN0000302710 |
| 1.77 | Rab28 | TRCN0000100697 |
| 1.75 | Rab30 | TRCN0000100402 |
| 1.74 | Rab11a | TRCN0000305864 |
| 1.70 | Rab1b | TRCN0000302711 |
| 1.69 | Rab1b | TRCN0000381762 |
| 1.65 | Rab36 | TRCN0000100804 |
| 1.65 | Rab31 | TRCN0000380682 |
| 1.65 | Rab7 | TRCN0000100881 |
| 1.63 | Rabl5 | TRCN0000257657 |
| 1.63 | Rab3c | TRCN0000089456 |
| 1.62 | Rab17 | TRCN0000100918 |
| 1.54 | Rab43 | TRCN0000318322 |
| 1.52 | Rab40c | TRCN0000054851 |
| 1.51 | Rab10 | TRCN0000335544 |
| 1.49 | Rab7 | TRCN0000100880 |
| 1.36 | Rab25 | TRCN0000100251 |
| 1.36 | Rab38 | TRCN0000102649 |
| 1.34 | Rab31 | TRCN0000100438 |
| 1.25 | Rab27a | TRCN0000100579 |
| 1.23 | Rab20 | TRCN0000102640 |
| 1.22 | Hps-1 | TRCN0000292556 |
| 1.20 | Rab15 | TRCN0000093173 |
| 1.18 | Rab32 | TRCN0000288450 |

**(B)** List of candidate genes

| **List of candidate genes/shRNA to validate** |  |  |
| --- | --- | --- |
| **Z- score** | **Gene** | **TRCN** |
| 2.60 | Rab35 | TRCN0000100532 |
| 2.07 | Rab25 | TRCN0000238207 |
| 1.86 | Rab37 | TRCN0000100818 |
| 1.83 | Rab10 | TRCN0000335543 |
| 1.69 | Rab1b | TRCN0000381762 |
| 1.65 | Rab31 | TRCN0000380682 |
| 1.54 | Rab43 | TRCN0000318322 |
| 1.52 | Rab40c | TRCN0000054851 |
| 1.49 | Rab7 | TRCN0000100880 |
| 1.25 | Rab27a | TRCN0000100579 |
| **1.22** | **Hps-1** | **TRCN0000292556** |
| **1.18** | **Rab32** | **TRCN0000288450** |
