# Supplementary material for: A Small-Scale shRNA Screen in Primary Mouse Macrophages Identifies a Role for the Rab GTPase Rab1b in Controlling Salmonella Typhi Growth
Source: Front Cell Infect Microbiol. 2021 Apr 7;11:660689. doi: 10.3389/fcimb.2021.660689 (PMC8059790; doi:10.3389/fcimb.2021.660689)
Supplement: Supplementary file 6 [file Table_5.docx]

**Table S5.** Primers used in this study

| **NAME** | **SEQUENCE (5´-3´)** |
| --- | --- |
| F-NGS | TCGTCGGCAGCGTCAGATGTGTATAAGAGACAGTCTTGTGGAAAGGACGA |
| R-NGS | GTCTCGTGGGCTCGGAGATGTGTATAAGAGACAGTCTACTATTCTTTCCCCTGCACTGT |
| F-Hps1 | TGGTTCGAGAATGACATGGGA |
| R-Hps1 | GGGTGGCTCTTGCTGTAGTAG |
| F-Rab1b | ATGAACCCCGAATATGACTACCT |
| R-Rab1b | TGCTGATGTAGCTCTCTGTGTA |
| F-Rab7 | AAGCCACAATAGGAGCGGAC |
| R-Rab7 | AGACTGGAACCGTTCTTGACC |
| F-Rab10 | GGCAAGACCTGCGTCCTTTT |
| R-Rab10 | GTGATGGTGTGAAATCGCTCC |
| F-Rab25 | GGGTTGAGTTCTCCACCCG |
| R-Rab25 | CCCCACGATAGTACGCAGA |
| F-Rab27a | TCGGATGGAGATTACGATTACCT |
| R-Rab27a | TTTTCCCTGAAATCAATGCCCA |
| F-Rab31 | GACACGGGGGTTGGGAAATC |
| R-Rab31 | ACAAGGCACGGTTTTGGTCA |
| F-Rab32 | CGTGGGTAAGACGAGCATCAT |
| R-Rab32 | CCCAGTTGAGAACTTTGAGGG |
| F-Rab35 | CCACAATCGGAGTGGATTTCA |
| R-Rab35 | CGTCGTAAACCACAATGACCC |
| F-Rab37 | CCAACCAGTCCTCTTTTGACAA |
| R-Rab37 | GCCTAGAAGCATAATCACCACG |
| F-Rab40c | GGGCAATAAGAATGATGACCCTG |
| R-Rab40c | TCCACATTGACGTTCTCCTTG |
| F-Rab43 | CAAGCTGGTGTTAGTGGGC |
| R-Rab43 | CCCAAATCTGTAACTTGACCCG |
| F-GAPDH | AGGTCGGTGTGAACGGATTTG |
| R-GAPDH | TGTAGACCATGTAGTTGAGGTCA |
